# Selected Topical Agents Used in Traditional Chinese Medicine in the Treatment of Minor Injuries- A Review

**DOI:** 10.3389/fphar.2016.00016

**Published:** 2016-02-05

**Authors:** Ping-chung Leung, Erik Chun-hay Ko, Wing-sum Siu, Ellie Suet-yee Pang, Clara Bik-san Lau

**Affiliations:** ^1^Institute of Chinese Medicine, The Chinese University of Hong KongHong Kong, Hong Kong; ^2^State Key Laboratory of Phytochemistry and Plant Resources in West China, The Chinese University of Hong KongHong Kong, Hong Kong; ^3^Centre for Clinical Trials on Chinese Medicine, Institute of Chinese Medicine, The Chinese University of Hong KongHong Kong, Hong Kong

**Keywords:** topical agent, herbal, fracture, fasciitis, tendonitis

## Abstract

Topical medicinal patches have been popular for the treatment of minor injuries like sprains and avulsions. Other inflammatory conditions like chronic musculo-tendinous pain and or fasciitis are also taken care of by local ointments or rubs. In the oriental communities, medicinal herbs frequently form the major components of the patches. In spite of the lack of scientific evidence of efficacy, the popularity of such traditional application persists for centuries. In this era of evidence-based clinical treatment, there is an urgent need to look into this traditional practice. The purpose should include a scientific verification of the efficacy of the practice, and once proven, further explorations would be indicated to bring the practice to a higher level. A system of comprehensive exploration was proposed and practiced in the past years to fulfill the aspiration. The research consisted of four areas:

(1) Identification of the suitable medicinal herbs for the topical study;

(2) Study of the biological activities of the selected herbs, concentrating on the areas of anti-inflammation, anti-oxidation, angiogenesis and cellular proliferation;

(3) Study on the transcutaneous transport of the chemicals of the selected herbs to deeper tissues; and

(4) Pilot clinical studies on common superficial inflammatory musculo-skeletal conditions to give objective clinical evidences to the topical applications.

Five herbs were identified as suitable candidates of study. They were put into relevant laboratory platforms and were proven to be anti-oxidant, anti-inflammatory and pro-angiogenic. Three of the herbs were prepared as topical patches with an enhancer and used to treat three common ailments in pilot clinical trials, viz., plantar fasciitis, undisplaced metatarsal fracture and tendonitis of the wrist (de-Quervain’s disease) and the elbow (Tennis elbow). The clinical results of the pilot studies were very positive. It is therefore concluded that further explorations are justified to create medicinal herb patches of even greater efficacy.

## Introduction

Centuries before the emergence of chemical drugs and pharmaceuticals, our ancestors have been making use of special plants for the control of disease symptoms and clinical problems. They could have followed the examples of animals which, by instinct, are capable of creating their primitive way to combat their odd infections and parasitic infestations (Campion, 1993). Successes of more or less similar applications among human beings have matured into various forms of Traditional Medicine which have been responsible for the maintenance of human health for centuries until more than one hundred years ago, when chemists started to systemically make use of small molecules of chemistry to deal with specific bodily ailments (Eisenberg et al., 1993).

During the long era of Traditional Medicine, the best known and probably the most widely practiced clinical offer has been herbal pastes for topical use (Pettman, 2007). The very early development of topical agents involving the use of medicinal herbs is natural and expected since external injuries to the musculo-skeletal system are most frequent. The long tradition of using topical herbal agents for musculo-skeletal injuries and pain has hence been well inherited and continued today, long after the maturation of pharmaceutical research. The sustained popularity of topical treatment, apart from the strong tradition inherited, must also be due to the convenience and subjective heart-felt efficacy (Yang, 2003). However, in spite of the many claims of efficacy, both from the manufacturers and users, subjective clinical feelings have not been supported by biological and pharmacological actions (Goldbeck et al., 1996). Whether the topical application does penetrate through the skin barrier to exert its pharmacological effects is equally unknown (Zhang et al., 2000).

## Research on Topical Agents

Since 10 years ago, the Institute of Chinese Medicine at The Chinese University of Hong Kong has engaged in a comprehensive study on topical herbal agents used for skeletal injuries. The scope of study included the selection of herbs; investigation on the biological effects of the herbs; the cross-skin barrier effects; and lastly the clinical evidences.

### Selection of Suitable Herbs for Topical Use

Study of old classics revealed that over 100 herbal items have been used as topical agents in the treatment of musculo-skeletal injuries (Yang, 2003; Chinese Pharmacopeia, 2005). How are we going to decide which are the effective ones to be chosen? The frequent appearances in classics, viz., the popularity, deserves special attention. In addition, the current concept of pain control and regeneration need particular attention. Isolated study reports on some of the herbs with regard to their anti-oxidant and anti-inflammatory properties would suggest that they could be suitable for the control of tissue oedema and pain. Similarly, those herbs shown to be promoting cell proliferations could be expected to help with tissue healing (Lundberg et al., 1997; Carano and Eilvaroff, 2003; Liao et al., 2005).

After a review of the classical records, five herbs were selected basing on the principles just described. They were Carthami Flos [*Carthamus tinctorius* L. (flower)], Dipsaci Radix [*Dipsacus asperoides* C.Y.Cheng & T.M.Ai (root)], Rhei Rhizoma [*Rheum officinale* Baill. (root and rhizome)], *Angelica Sinensis* Radix [*A. sinensis* (Oliv.) Diels (root)] and Achyranthis Bidentatae Radix [*Achyranthes bidentata* Blume (root)]. Carthami is well known for its antioxidant effects. Dispaci has been described as an “bone repairing” agent. *A. Sinensis*, Achyranthis Bidentatae and Rhei have been studied for their anti-inflammatory and neo-vascularisation effects (Song, 2007; Peng et al., 2010; Zhou et al., 2014). Subsequently only three herbs were used in the study.

Traditionally many herbs are mixed together to achieve a synergistic combination. We do not advocate the use of more than 3–5 herbs beyond which quality control of the selection would become more difficult (Leung, 2015). The major constituents of the selected herbs are known and their HPLC data are available in the Chinese Pharmacopeia (2005).

Proper vouchering of sample herbs are standard practices in order to ensure sustainable development (Bye and Botanico, 1986).

### Platform Studies on the Biological Effects of the Herbs

Platform laboratory studies to confirm the biological properties of the chosen herbs, included their anti-oxidant, anti-inflammatory and proangiogenic effects, tested alone and later, in combination. *In vitro* studies gave convenient judgments on the three essential properties. Using RAW264.7 cell line to suppress the NO production demonstrated the antioxidant effects; while the promotive effects on HUVEC and UMR106 cells confirmed the anti-inflammatory and vascular proliferative effects (Peng, 2009; Peng et al., 2010; Siu et al., 2015). The promotion of osteoblastic proliferation could be demonstrated *in vitro* using animal osteoblast cultures (Feng et al., 2004). *In vivo* tests included sophisticated angiogenic studies using the zebra fish embryo model (Zhou et al., 2014). A complicated bone fracture model was built on the rabbit and rat, which allowed radiological assessment on the healing as well as concomitant serological changes (Leung et al., 2010). Mechanistic details of the biological effects of the herbs have been worked out by partners in our group (Peng et al., 2010; Siu et al., 2015). All animal experiments were approved by the animal experimentation ethics committee at the Chinese University of Hong Kong.

### Confirmation of Transcutaneous Transport of Chemical Marker Material Across the Skin Barrier to Achieve its Direct Pharmacological Effects on the Injured and Inflamed Tissues

*In vitro* and *in vivo* testings of transcutaneous drug transport are essential steps to confirm the efficacy of topical agents. Special device like the Franz Diffusion cell gives *in vitro* information about the diffusion of medicinal agents through a selected membrane, either artificial or prepared from the skin of an animal (Zhao, 2004). *In vivo* testings would involve the appearance of the recognized chemical marker of the medicinal agent being used, either within the subcutaneous tissue or in the circulation of the animal used for study (Peng, 2009).

When the topical agent consists of a simple chemical compound, diffusion study is not complicated because a direct analysis of the transfer of the compound would fulfill the requirement. To study the transport of herbal materials across a membrane or skin, complex chemicals are involved. Authentication of commonly used medicinal plants is dependent on the identification of specific chemical markers for a particular plant. This practice is obviously a compromise since the plant contains numerous chemicals rather than a single or a few compounds. However, before a better method can be developed, the appearance of a known chemical marker of a specific medicinal plant across a membrane, could be considered an objective proof about the transfer, although it is only a qualitative and partial demonstration of the delivery.

All the five medicinal plants chosen for this study have officially recognized chemical markers and five of them are selected for the *in vitro* and *in vivo* studies. Using the Franz diffusion chamber with either an artificial or mouse skin membrane, the markers were identified in the receptor compartment, thus proving the across membrane transport (Zhao, 2004).

Traditionally topical herbal preparations make use of enhancers to facilitate the quality and quantity of skin transfer. Borneol has been the most popular agent used for this purpose in traditional Chinese medicine. Alternatively, a pharmacological agent, ozone, has been a popular enhancer used in the pharmaceutical industry with proven facilitating effects (Chen et al., 1995; Chen and Wang, 2004).

Studying the bioavailability of orally consumed herbal preparation is a difficult challenging job since the intraluminal metabolic activities occurring in the small and large bowels could be affecting the herbal substances in the most complicated manners. For the topical treatment, once penetration could be proven, the drug effects could be much similar to the *in vitro* studies.

In our study, penetration was further verified using live animals when the serum was taken for the analysis of the marker chemicals after the topical application. Small quantities of marker chemicals were demonstrated in the serum which objectively indicate the presence of the herbal substance within the site of application (Peng, 2009).

### Collecting Quality Clinical Evidences

Since topical agents are widely used in minor injuries resulting in pain, swelling and loss of function, testing any new innovative topical agent could follow this general direction. In the hospital practice of traumatology and accident-emergency management, where injuries are usually more serious, topical treatment is seldom considered. However, topical treatment still retains its popularity under situations of minor injury or persistent pain and swelling in spite of standard treatment.

A number of common limb injuries presenting as painful and inflammatory conditions were chosen for the study of this topical herbal agent. The conditions involve the inflammation of tendons and fascia. The three conditions chosen were:-

(a) Undisplaced fracture of the distal metatarsal bone. Swelling and pain commonly persist in spite of fracture healing. Topical agent to consolidate the bone healing and to better control the symptoms of pain and swelling is a logical consideration.
(b) Plantar fasciitis in the foot classically lead to long lengthy duration of heel pain which affects walking and standing. Topical treatment over the heel was chosen as another study target.
(c) In the upper limb, tendon inflammation like de Quervains disease of the short extensor and abductor of the thumb and wrist extensor origin tendonitis (tennis elbow) are common conditions suitable for clinical study of topical application.

Henceforth, three pilot studies were organized to serve as “proof of concept” trials in preparation for larger scale studies in future. The study design followed the standard requirements for similar clinical trials so that proper clinical data could be built up to be compared with other options of treatment. The study protocols followed principles recommended by the Declaration of Helsinki and had been approved by the hospital ethical committee. The methodology of study and results of treatment are presented in the **Table 1**. The topical treatment for all three sites had been very well accepted by patients. Recurrences after cessation of local treatment were observed in some cases, but the intensities were apparently much less than before treatment (Tse et al., 2015a,b).

**Table 1:** Summary of three pilot studies using the topical herbal application.

Clinical condition	Fifth Metatarsal fracture Tse et al., 2015a	Plantar fasciitis Tse et al., 2015b	De Quervain’s and Tennis Elbow
Aim	Whether topical herbal formula helps to reduce pain/facilitate healing	Whether topical herbal formula helps to reduce pain	Whether topical herbal formula helps to reduce pain
Methodology	A pilot, open label, one arm self-controlled, observational study	A pilot, open label, one arm self-controlled, observational study	A pilot, open label, one arm self-controlled, observational study
Study duration	Six weeks or pain/swelling disappear	Six weeks or pain/swelling disappear	Six weeks or pain/swelling disappear
Number of subjects	10	9	De Quervain’s 5 Tennis Elbow 6
Inclusion criteria	Acute traumatic fracture of fifth metatarsal No displacement	Plantar fasciitis (Heel pain) 18–65 years. History over 4 weeks	History over 4 weeks
Exclusion criteria (Apart from pregnancy, breast feeding, TCM sensitivity)	Open injury	Ulceration	Acute injury
Study product	Formula : semi solid paste containing concentrate of three herbs extracts + Borneol	Same formula	Same formula
Assessments	Interview, orthotics •American Orthopedic Foot and Ankle Society + Ankle Hindfoot Scale (AOFAS) •Foot/Ankle Ability Measure (FAAM) •Pain evaluation – visual analog •Ultrasonic study •Check blood for inflammation cytokine •Water displacement •X ray site •3D Scan Gogh II imaging	Interview, map site of pain •Foot function index •Pain evaluation – visual analog •Ultrasonic study •Check blood for inflammation cytokine	Interview, Map site of pain •Disabilities of the arm, shoulder, and head •Pain evaluation – visual analog •Ultrasonic study
Treatment	Apply patch over fracture site, change every 3 days until 6 weeks Data checking 0 and 6 weeks	Apply patch over heel, change daily until 6 weeks Data checking 0 and 6 weeks	Apply patch over the wrist at the base of the thumb for de Quervain’s and outer side of the elbow joint for tennis elbow. Change daily until 6 weeks Data checking 0 and 6 weeks
Outcome measure	Swelling assessment/fracture healing	Pain relief Inflammation control	Pain relief Inflammation control
Results:			
Safety/Allergy	No serious adverse effects	No serious adverse effects	No serious adverse effects
Pain evaluation visual analog scale	28% decrease in morning pain (at 2 weeks vs. baseline) 41% decrease in evening pain (4 weeks vs. baseline)	56% decrease in morning pain (at 4 weeks vs. baseline)	de Quervain’s 52% decrease in morning pain (6 weeks vs. baseline) 53.8% decrease in evening pain (6 weeks vs. baseline) Tennis Elbow 47.8% decrease in morning pain (6 weeks vs. baseline) 52% decrease in evening pain (6 weeks vs. baseline)
Ultrasound assessment	Para-fracture oedema 20% reduction	Fascia thickness 9.07% reduction	Oedema reduction positive

## Discussions

Transdermal topical drug delivery offers a non-invasive route of drug administration. Ten years of large scale explorations on the clinical science of traditional herbal medicine applied as topical agents for anti-inflammatory and pain control measures, using standard laboratory platforms and small scale pilot clinical studies, have given us confidence that the traditional method of treatments does have objective evidences of efficacy. Established surgeons and physicians should refrain from teasing topical agents as “myths,” “counter-irritants” or “ritual practices.” Topical agents providing anti-oxidant and anti-inflammation effects on the diseased tissues after penetrating the skin barrier is now a proven fact. The vascular promotion ability observed further supports the revascularization and regeneration of tissues accordingly. On the other hand, users and advocates should not dogmatize the old view that the topical agent heals perfectly on its own. Instead, the topical agent helps to control symptoms, whereas the primary causes of the pain and swelling could be the result of more complicated pathologies that requires the joint efforts of caring teams to achieve the best clinical outcome (Hung et al., 2015).

The common conditions studied had known predisposing causes of chronic stress and/or repetitive minor injuries. To ensure cure and prevention of recurrence, the primary causes must be dealt with at the same time. Without taking effective care of the causative mechanisms relief of the symptoms could be transient. Topical agent administration therefore needs to go hand in hand with active rehabilitation measures.

Further work needs to be done so as to give more comprehensive understanding of the mechanisms of action of the herb extracts. The molecular aspects of the biological effects need to be explored so that more suitable medicinal herbs could still be identified and either small chemical molecules or groups of chemicals from the gross extracts of the herbs could be used to give more potent effects.

On the skin surface penetration, particle sizes manipulation has been the many innovations of the nanotechnology experts. The application of nanotechnology would be able to enhance penetration, while at the same time ensure that the ideal quality and quantity of the chemical components with desirable effect be adequately delivered (Chan et al., 2009).

## Author Contributions

PL had responsibility for all parts of the manuscript. WS, EK, and CL had worked on the herbs biological and mechanistic and pre-clinical data mentioned in the manuscript.

## Conflict of Interest Statement

The authors declare that the research was conducted in the absence of any commercial or financial relationships that could be construed as a potential conflict of interest.
